# Patients in a private hospital in India leave the emergency department against medical advice for financial reasons

**DOI:** 10.1186/1865-1380-7-13

**Published:** 2014-02-25

**Authors:** Sassan Naderi, John R Acerra, Kathryn Bailey, Pinaki Mukherji, Taraknath Taraphdar, Tirtha Mukherjee, Abin Pal, Mary Frances Ward, Kathryn Miele, Maxwell Mathias, Richard Tan, Myriam Kline, Kumar Alagappan, Robert Silverman

**Affiliations:** 1 North Shore-LIJ Health System, 300 Community Drive, Manhasset, NY 11030, USA; 2 Department of Emergency Medicine, Long Island Jewish Medical Center, 270-05 76th Avenue, New Hyde Park, NY 11040, USA; 3 Mission Hospital Iman Kalyan Sarani, Sector IIC Bidhan Nagar, Durgapur, Burdwan, West Bengal 713212, India

**Keywords:** Against medical advice, Discharge, Emergency department, Emergencies, International emergency medicine, India

## Abstract

**Background:**

Some reports indicate financial concerns as a factor affecting ED patients leaving the acute care setting against medical advice (AMA). In India, no person is supposed to be denied urgent care because of inability to pay. Since a large proportion of the Indian health care system is financed by out-of-pocket expenses, we investigate the role of financial constraints for ED patients at a private hospital in India in leaving AMA.

**Methods:**

A prospective ED-based cross-sectional survey of patients leaving AMA was conducted at a private hospital in India from 1 October 2010 to 31 December 2010. Descriptive statistics and the chi-square test were used to identify associations between financial factors and the decision to leave the hospital AMA.

**Results:**

Overall, 55 (3.84%) ED patients left AMA, of which 46 (84%) reported leaving because of financial restrictions. Thirty-nine (71%) respondents indicated the medical bill would represent more that 25% of their annual income. Females (19/19) were more likely to leave AMA for financial reasons compared to males (27/36, *p* = 0.017). Among females who signed out AMA, the decision was never made by the female herself.

**Conclusion:**

The number of people leaving the ED AMA in a private Indian hospital is relatively high, with most leaving for financial reasons. In most cases, women did not decide to leave the ED AMA for themselves, whereas males did. This survey suggests that steps are needed to ensure that the inability to pay does not prevent emergent care from being provided.

## Background

With nearly 1.2 billion people, India is one of the most populous countries in the world [1]. India’s rapidly growing economy has expanded its role in the global marketplace, although the development of its health system appears to be lagging behind its economic development [1]. The total expenditure on health per capita is $132 in a country where the gross national income per capita is only $2,930 [2]. According to the World Health Organization (WHO), newly emerging non-communicable diseases associated with an improving standard of living are adding further strain to the health system, along with the persistence of communicable diseases [2-4]. In addition, there is wide variability in the wealth distribution, demographics, and politics, which also influences access to health care [5].

The Indian health care system is a complex integration of the national public health system, private hospitals, and alternative medicine practitioners [3,4]. Approximately 70-80% of health spending nationwide is from individual out-of-pocket payments [5,6]. The public health care system is free for people below the poverty line, yet despite the demands on this system, it lacks resources and accountability [3]. India’s public health system constitutes only 1% of its total gross domestic product, placing it below most low-income countries and in the bottom 20% of all countries [6]. Because the public health system is perceived to be poorly equipped to provide quality care, the majority of Indians seek health care in the private system and pay out-of-pocket [3,5].

As outlined in Article 21 of the Constitution of India, the State is obliged to safeguard the right to life of every person, and the judicial system mandates the delivery of timely medical treatment by all hospitals regardless of a patient’s ability to pay or their medico-legal status [7-9]. This mandate is similar to the Emergency Medical Treatment and Labor Act in the US, which protects patients from being turned away from hospitals because of their inability to pay for care [10]. However, considering the cost of care at private hospitals and the limited availability of free care for the poor in the public system, it is not clear whether patients emergently presenting to a private facility are able to obtain acute care or whether they leave an acute care facility without treatment because of economic constraints. Often, patients presenting to a private emergency department have initial cardiac and respiratory stabilization performed, but further management cannot continue if patients are unable to pay for services.

Leaving the emergency department without treatment or against medical advice (AMA) is associated with poor outcomes, including an increased risk for mortality and readmission [11,12]. Among wealthier nations, there is a wide range of reasons that patients sign out AMA, while in a limited number of studies from poorer nations financial constraints are the more common cause. In two Nigerian hospitals, research found that 29-33% of patients leaving AMA left for financial reasons [13,14]. No studies to date have assessed the reasons why patients in India leave the ED AMA. Based on limited data from other developing countries and the infrastructure of the Indian health care system, we hypothesize that financial constraints will be an important reason for refusing emergent care in a private hospital in India.

## Methods

This was a prospective ED-based cross-sectional study of patients leaving AMA and was conducted at a private hospital with approximately 10,000 ED visits per year in eastern India. At the study site, most patients visiting the hospital have some form of private health insurance that covers the cost of admissions in the wards and intensive care unit but does not cover the cost of ED care. An anonymous, 13-item survey, available in English and the predominant local language, was given to a convenience sample of patients who left the ED AMA from 1 October 2010 to 31 December 2010. The survey included patient diagnosis as well as demographic information such as patient religion, age, gender, and occupation. It also included questions concerning leaving AMA, some of which are summarized in Table 1. Potential subjects were excluded if they were unable to communicate in the language of the survey (verbal and written), had an overt psychiatric illness preventing them from completing the study, left the ED before the completion of the survey, were a prisoner, or declined participation. Patients filled out the survey anonymously and dropped it in a box in the emergency department. Those who were unable to read or write could request assistance from staff members who were not study investigators to read the survey questions to them and record their responses. The survey was conducted with the patients alone unless they specifically requested a family presence. IRB approval was obtained from the Investigational Review Board of the North Shore-LIJ Health System with a written agreement from the participating hospital establishing the collaboration. The sample size was based on availability of surveyors to obtain information at the hospital.

**Table 1:** Select survey questions and potential responses

**Survey questions**	**Multiple choice responses**
Why did you sign out against medical advice?	• Financial restrictions
	• Time constraints
	• Dissatisfaction with service
	• Long wait time
	• No hope of improvement in my illness
	• Emotional stress at home
	• Child care
What is your annual income?	• Under Rs. 50,000
	• Rs. 50,000 % Rs. 1,00,000
	• Rs. 1,00,000 % Rs. 2,50,000
	• Above Rs. 2,50,000
What percent of your annual income do you think this bill will represent?	• Under 10%
	• 10% % 25%
	• More than 25%
Do you have health insurance?	• Yes
	• No
Will you seek medical attention at another medical facility	• Yes
	• No
Who is deciding to leave?	• Self
	• Family
	• Friends
	• Others
Did the doctors explain the consequences of leaving against medical advice?	• Yes
	• No

Descriptive statistics were computed using standard methods for proportions and means, and the chi-square (or Fisher’s exact) test was used to determine whether there was a significant association between categorical variables. The primary endpoint variable was the relationship between personal income and signing AMA, and in addition other factors that influence a patient’s decision to leave the emergency department AMA were evaluated.

## Results

Among the 1,854 patients seen in the ED during the study period, 71 (3.8%) left the hospital AMA, and of the 71 patients who left AMA, a total of 55 (71.4%) completed the survey. The overall average age of patients who left AMA was 41 years, and five patients were under 18 years with four of the five under 1 year of age. There were 36 (65%) male respondents, and the majority of respondents were Hindu (51, 92%). The range of diagnoses were diverse, with infectious disease (13, 24%) and trauma (13, 24%) the most common (Table 2). The majority of the 55 respondents were homemakers (12, 22%), students (10, 18%), and service workers (10, 18%), with other patients being laborers, retired, and professionals. Fifty-three (96%) study participants stated that they would seek medical attention elsewhere, and all respondents stated that doctors did explain the consequences of leaving AMA.

**Table 2:** Diagnosis of patients leaving AMA

**Diagnosis**	**Number of patients (%)**
Trauma^1^	13 (24%)
Infectious disease^2^	13 (24%)
Surgical/gastrointestinal^3^	10 (18%)
Neurologic^4^	7 (13%)
Cardiac^5^	5 (9%)
Pulmonary^6^	4 (7%)
Psychiatric	4 (7%)

^1^For example, long bone fracture, head injury.

^2^For example, pneumonia, fever, sepsis, pyelonephritis.

^3^For example, appendectomy, gastroenteritis, pancreatitis, colecistitis, rectal bleed, and dyspepsia.

^4^For example, CVA, seizure, intracranial hemorrhage.

^5^For example, ACS, chest pain (NOS).

^6^For example, COPD, hemoptysis.

**Figure 1:**
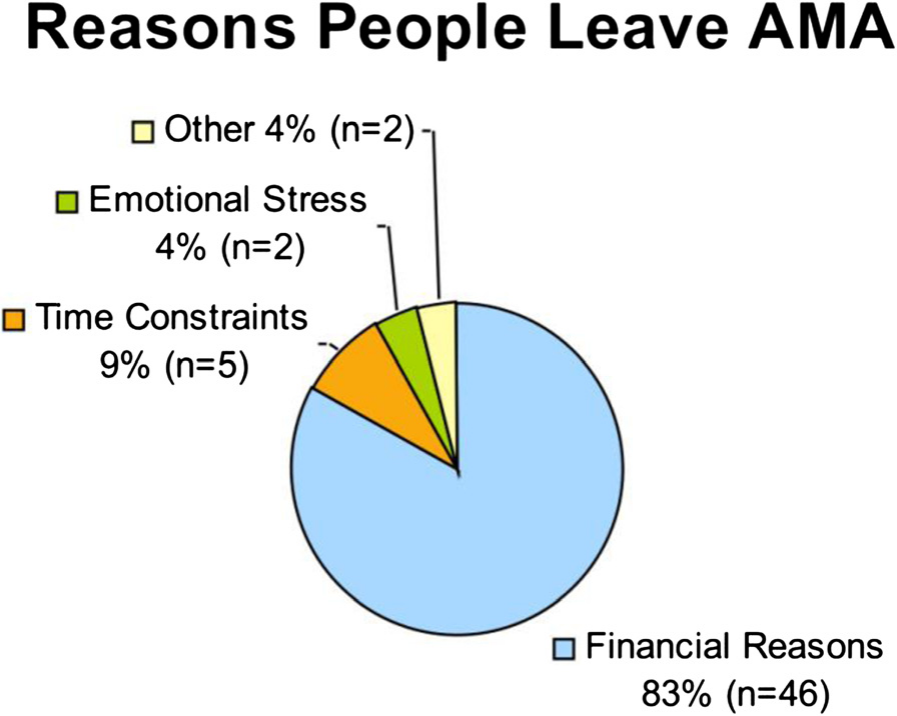
Reasons why people leave the hospital against medical advice (AMA) at a private hospital in West Bengal, India.

The primary reason given by 46 (84%) respondents for leaving AMA was financial restrictions (Figure 1). A total of 39 (71%) indicated the medical bill for that visit would represent more than 25% of their annual income, and 16 (29%) indicated it would be 25% or less than their annual income. Patients with lower annual incomes left for financial restrictions a greater percentage of the time compared to patients with higher annual incomes: 8 of 8 (100%) respondents with an annual income of less than 50,000 rupees (~$1,100 USD) left AMA; 18 of 20 (90%) with between 50,000 rupees and 100,000 rupees (~$2,200 USD) left AMA, and 20 of 27 (74%) with above 100,000 rupees left AMA (*p* = 0.04). Forty-four (80%) of those surveyed reported not having private insurance. Among the 11 patients who had private insurance, 8 (73%) identified financial restrictions as the primary reason for leaving AMA, and 38/44 (86%) of those without private insurance left for financial reasons.

Respondents identified who was responsible for the decision to leave AMA. The majority (36, 65%) indicated that a family member made the decision to leave, and 35/36 (97%) of those individuals also reported leaving AMA because of financial restrictions. There were 16 respondents who indicated making their own decision to leave AMA; all 16 were male. None of the female respondents made the decision to leave AMA. In addition, females more frequently left for financial reasons than males (19/19 vs. 27/36, *p* = 0.017).

## Discussion

In this survey conducted in a private Indian emergency department, we found the primary reason for signing out AMA was difficulty paying for acute medical care. As noted in a consensus statement by the Association of Physicians in India, private hospitals typically offer services on a fee-paying basis including emergency care, and payment for testing and physician services is required before services are rendered [15]. In critical situations, a private hospital may perform initial stabilization, but then families must decide whether to pay for further services, transport the patient to a government hospital, or forego further care [15]. Advanced payment in private facilities appears to have been the barrier in obtaining care for most of the patients in our study who left the ED without treatment.

A total of 3.8% of patients presenting to the emergency department left AMA, a number higher than the 1.3% cited as leaving from a nationwide report in the USA [16]. A number of studies from countries across the globe have reported on reasons for leaving AMA. In the US patients give multiple reasons for signing AMA, including disagreement with the medical plan, feeling better, personal obligations, financial constraints, and drug-seeking behavior [17,18]. In a hospital in Hong Kong, the two most cited reasons for leaving AMA were wanting to observe symptoms at home or for â€˜personal reasons;’ in this setting, medical fees were nominal, and financial reasons were not cited as a reason for leaving [19]. These findings differ from those in developing countries, where leaving for financial reasons from the ED and hospital was found to be more common [13,14]. The frequency of leaving the ED of the current study hospital AMA for financial reasons was among the highest found in the literature.

In our study, a greater percentage of those with lower annual incomes left AMA compared to those with higher incomes, with most patients indicating that the current hospital bill would have exceeded 25% of their annual income. Although laws in India indicate life-saving treatments must be offered regardless of ability to pay, these are unfunded mandates, and the burden of free emergency care falls on the provider or hospital. This would appear to be a substantial obstacle for obtaining care in non-governmental facilities.

Although financial reasons were the most often cited for leaving AMA, this was even more apparent in women compared to men. A number of gender-related social issues have been identified in India where, according to a global study conducted by Thomson Reuters, women are identified to be among the most vulnerable in the world for reasons that include access to health care [20]. It has been found that contributions Indian women make to families are often overlooked, and instead women are viewed as economic burdens [21]. In India, the paternalistic family structure typically has men more often making the decision to leave the hospital AMA for themselves, whereas women typically defer these decisions to their families. This may not be in the best interest of female patients as their medical decision makers may not understand the complexity of the medical situation at hand and could make choices that jeopardize patients’ health.

Among the study limitations is the single-site data collection at a private hospital. Although we cannot determine if the findings are generalizable to all emergency departments in India, the practice of up-front payment at this hospital appears typical for private hospitals in India [15]. In addition, we did not follow patients up after leaving the ED to determine whether they sought care elsewhere or the health-related consequences of leaving AMA. Expansion to a multisite study that includes public, private, and emergency departments of varying sizes in India will broaden the scope our findings.

## Conclusion

The proportion of people leaving the ED AMA in a private Indian hospital is high, and most leave the hospital for financial reasons. In most cases, the family of the patient made the decision to leave the hospital AMA. This survey suggests that steps are needed to ensure that the inability to pay does not prevent emergent care from being provided. Future studies on the provision of emergency care that include multiple hospitals in India and patient outcome data should be done to further describe the impact on the health of the Indian population.

## Competing interests

There are no competing interests among the investigators of this study.

## Authors’ contributions

SN, JA, PM, MW, KA, TT, TM, AP and RS participated in study design and data collection. MK carried out the statistical analysis of the data. KB, KM, MM, and RT participated in study design and coordination and helped draft the manuscript. All authors read and approved the final manuscript.
